# Supplementary material for: Recognition of intracranial hypertension using handheld optical coherence tomography in children (RIO Study): a diagnostic accuracy study protocol
Source: BMJ Open. 2022 Jan 11;12(1):e048745. doi: 10.1136/bmjopen-2021-048745 (PMC8753392; doi:10.1136/bmjopen-2021-048745)
Supplement: Author's manuscript [file bmjopen-2021-048745.draft_revisions.pdf]

BMJ Open is committed to open peer review. As part of this commitment we make the peer review history of every article we publish publicly available.

When an article is published we post the peer reviewers' comments and the authors' responses online. We also post the versions of the paper that were used during peer review. These are the versions that the peer review comments apply to.

The versions of the paper that follow are the versions that were submitted during the peer review process. They are not the versions of record or the final published versions. They should not be cited or distributed as the published version of this manuscript.

BMJ Open is an open access journal and the full, final, typeset and author-corrected version of record of the manuscript is available on our site with no access controls, subscription charges or pay-per-view fees (http://bmjopen.bmj.com).

If you have any questions on BMJ Open's open peer review process please email info.bmjopen@bmj.com

BMJ Open

Recognition of Intracranial Hypertension using Handheld Optical Coherence Tomography in Children (The RIO Study): Protocol

Journal:	*BMJ Open*
Manuscript ID	bmjopen-2021-048745
Article Type:	Protocol
Date Submitted by the Author:	05-Jan-2021
Complete List of Authors:	Rufai, Sohaib; Great Ormond Street Hospital For Children NHS Foundation Trust, Clinical and Academic Department of Ophthalmology; University of Leicester, University of Leicester Ulverscroft Eye Unit<br>Jeelani, Noor ul Owase; Great Ormond Street Hospital For Children NHS Foundation Trust, Department of Neurosurgery<br>Bowman, Richard; Great Ormond Street Hospital for Children NHS Foundation Trust, Clinical and Academic Department of Ophthalmology<br>Bunce, Catey; Royal Marsden Hospital NHS Trust, Clinical Trials Unit<br>Proudlock, Frank; University of Leicester, University of Leicester Ulverscroft Eye Unit<br>Gottlob, Irene; University of Leicester, University of Leicester Ulverscroft Eye Unit
Keywords:	OPHTHALMOLOGY, Paediatric neurosurgery < PAEDIATRIC SURGERY, Paediatric ophthalmology < OPHTHALMOLOGY, Paediatric plastic & reconstructive surgery < PAEDIATRIC SURGERY, Neuro-ophthalmology < NEUROLOGY, Paediatric neurology < PAEDIATRICS

SCHOLARONE™
Manuscripts

RIO Study Protocol

Title Page

Title: Recognition of Intracranial Hypertension using Handheld Optical Coherence Tomography in Children (The RIO Study): Protocol

Short title: RIO Study Protocol

Authors: Sohaib R. Rufai (BMBS, MRes)[1-3], Noor ul Owase Jeelani (MPhil, FRCS)[2], Richard Bowman (MD, FRCOphth)[1], Catey Bunce (MSc, DSc)[4], Frank A. Proudlock (MSc, PhD)[3], Irene Gottlob (MD, FRCOphth)[3]

Affiliations:

1. Clinical and Academic Department of Ophthalmology, Great Ormond Street Hospital for Children NHS Foundation Trust and UCL Great Ormond Street Institute of Child Health, London, United Kingdom.
2. Craniofacial Unit, Great Ormond Street Hospital for Children NHS Foundation Trust and UCL Great Ormond Street Institute of Child Health, London, United Kingdom.
3. The University of Leicester Ulverscroft Eye Unit, Leicester Royal Infirmary, Leicester, United Kingdom.
4. Clinical Trials Unit, The Royal Marsden NHS Trust, London, United Kingdom.

Corresponding author: Professor Irene Gottlob (MD, FRCOphth); Correspondence address: The University of Leicester Ulverscroft Eye Unit, Leicester Royal Infirmary, Robert Kilpatrick Clinical Sciences Building, PO Box 65, Leicester LE2 7LX, United Kingdom; E-mail: ig15@leicester.ac.uk; Tel: +44 (0)116 2523152; Fax: +44(0)116 2231996

Sponsor: University of Leicester

Sponsor Reference Number: 0348

Ethics Reference: 12/EM0261

Protocol Date: 01/11/2019

Competing Interests Statement: Dr. Proudlock reports personal fees from Leica Microsystems to run a virtual clinical symposium for hand-held optical coherence tomography, outside the submitted work. Dr. Rufai, Dr. Jeelani, Dr. Bowman, Dr. Bunce and Prof. Gottlob have no competing interests to report.

Data Availability Statement: No data are available as this is a study protocol.

Funding Statement: This work is supported by the Medical Research Council, UK (Grant No. MR/N004566/1). Dr Sohaib Rufai is funded by the National Institute of Health Research Doctoral Fellowship Award. This work is supported by the NIHR Great Ormond Street Hospital Biomedical Research Centre. This protocol proposes independent research funded by the National Institute for Health Research (NIHR) and the Medical Research Council, UK. The views expressed are those of the author(s) and not necessarily those of the MRC, the NHS, the NIHR or the Department of Health and Social Care.

For peer review only - http://bmjopen.bmj.com/site/about/guidelines.xhtml

For peer review only

RIO Study Protocol

Abstract

Introduction

Paediatric intracranial hypertension (IH) is a rare but serious condition that can pose deleterious effects on the brain and vision. Estimating intracranial pressure (ICP) in children is difficult. Gold standard direct ICP measurement is invasive and impractical to be routinely employed with repeated measures for all children at risk. This study proposes to assess the diagnostic accuracy of handheld optical coherence tomography (HH-OCT), a non-invasive imaging method, to detect IH in children.

Methods and analysis

This is a single-centre prospective study evaluating the diagnostic accuracy of handheld OCT for IH in at risk children. Inclusion criteria include clinical and/or genetic diagnosis of craniosynostosis, IIH, space occupying lesion or other conditions association with IH and age 0-18 years old. Exclusion criteria include patients older than 18 years of age and/or absence of condition placing the child at risk of IH. The primary outcome measures are handheld OCT and 48-hour ICP assessments, which will be used for diagnostic accuracy testing (sensitivity, specificity, positive predictive value, negative predictive value and accuracy). Main secondary outcome measures include visual acuity, fundoscopic examination, contrast sensitivity, visual field testing and visual evoked potentials, wherever possible.

Ethics and dissemination

Ethical approval was granted for this study by the East Midlands Nottingham 2 Research Ethics committee (UOL0348/IRAS 105137). Our findings will be disseminated through presentation at relevant meetings, peer-reviewed publication and via the popular media.

Trial registration number: ISRCTN52858719

Strengths and limitations of this study

- To the best of our knowledge, this the first study to harness the potential of handheld optical coherence tomography (OCT) in the detection of intracranial hypertension (IH) in at risk children.
- *In vivo* handheld OCT images can be acquired in awake children during routine clinical appointments.
- The study findings may optimise management of children at risk of IH.
- This study protocol should promote transparency of our methodology and reduce the risk of duplication.
- The main limitation could be low patient numbers due to rarity of paediatric IH, albeit we aim to achieve a powered cohort.

For peer review only - http://bmjopen.bmj.com/site/about/guidelines.xhtml

Introduction

Intracranial hypertension

First described by Quincke in 1896, intracranial hypertension (IH) remains an area of major importance among the medical profession.[1] If undetected and untreated, IH can pose devastating consequences including visual impairment, cognitive impairment, disability and even death.[2, 3] This is of particular significance in subacute conditions affecting children, because insidious elevation of intracranial pressure (ICP) may only be detected after insult to the brain and vision has already begun.[2, 4] Therefore, it is crucial that IH is detected and treated as early as possible to prevent or limit the sequelae of IH.

The incidence of IH is approximately 0.63-0.71 per 100,000 children.[5, 6] IH can be categorised as primary (or idiopathic) and secondary; the former is characterised by elevated ICP with no identifiable cause, while the latter is characterised by elevated ICP directly resulting from another condition. [4, 7] Common associations of primary IH include female sex, high body mass index (BMI) and postpubertal status, however it can occur in children with no predilection for sex or high BMI.[4, 8-14] Examples of secondary IH include expansive skull pathology, i.e. space occupying lesion or hydrocephalus, or constricting skull pathology, such as craniosynostosis, characterised by premature fusion of cranial sutures.[2-4]

Measuring intracranial pressure

Measuring ICP in children presents a number of challenges. The gold standard method is direct intraparenchymal measurement, but this involves hospital admission and carries significant risks including infection, bleeding, CSF leak, mechanical failure and general anaesthesia risks.[15] Another limitation of intraparenchymal measurement is the difficulty in obtaining repeated measures over time. Furthermore, there is no clinical consensus on timing, frequency and duration for accurate measurement, or what figure constitutes raised ICP in children. Many consider ICP greater than 15mmHg to be elevated[2, 16-24], but Hayward *et al*[25] argue this could reasonably be raised to 20 mmHg.[25] Overnight ICP monitoring during natural sleep, which provides a ‘‘grand average’’ with description of the dynamics of the ICP, is considered the ‘‘gold standard’’.[26]

The ideal measure of ICP would be non-invasive, acceptable for children, quantitative and would permit serial measurements to appreciate evolving changes related to rising ICP. Existing surrogate measures of ICP do not fulfil all these criteria, including fundoscopy[27], radiography[28] and ocular ultrasonography[29], each failing to provide adequate sensitivity to be used as screening tools. In particular, fundoscopy is routinely used in paediatric ophthalmology clinics to detect IH. Tuite *et al*[27] demonstrated that fundoscopy yielded sensitivity of only 22% for IH in children with craniosynostosis aged under 8 years old, while specificity was 98%. Hence, papilloedema detected on fundoscopy reliable indicates IH in younger children with craniosynostosis, but crucially its absence does not exclude IH.

Optical coherence tomography

The optic nerve is primarily intracranial, hence IH can cause optic nerve swelling with secondary retinal changes. Optical coherence tomography (OCT) is a non-invasive imaging modality that can rapidly produce ultra-high resolution cross-sectional images of the optic nerve and retina *in vivo*.[30] OCT scans of the optic nerve head can provide highly reproducible quantitative measures associated with IH within seconds.[31]

For peer review only - http://bmjopen.bmj.com/site/about/guidelines.xhtml

RIO Study Protocol

Our recent systematic review[32] revealed three studies[16, 33, 34] evaluating OCT to detect IH in children with craniosynostosis. There was a lack of evidence to formally recommend OCT to detect IH in this setting. Swanson *et al*[16] compared OCT parameters to single intra-operative ICP measurements, demonstrating overall sensitivity of 89% (95% confidence intervals (CI): 62-94%) and specificity of 62% (95% CI: 41-79). However, no study has used the gold standard ICP measurement achieved by overnight ICP monitoring.[26] OCT devices used in these studies included Spectralis[33,34] (axial resolution 3.9µm; Heidelberg Engineering, Heidelberg, Germany) and iVue (axial resolution 5µm; Haag-Streit, Wedel, Germany).[16] The former was used in older, awake children[33, 34] whilst the latter was used in younger children under general anaesthesia.[16]

Handheld optical coherence tomography

OCT has revolutionised the clinical practice of ophthalmology over the past two decades. However, infants have been largely deprived of this technology as conventional OCT machines are designed for adults, requiring compliance with a chin rest, good fixation and cooperation. These limitations have been overcome with the recent advent of the handheld spectral-domain OCT designed for infants, which is demonstrably reliable in awake infants.[30, 35] Normal development of the optic nerve[36] and retina[37] in children has recently been described using handheld OCT. A wide range of conditions have also been investigated using handheld OCT in children, including retinopathy of prematurity[38], nystagmus[35], foveal hypoplasia[39], optic nerve hypoplasia[40], achromatopsia[41], albinism[42], primary congenital glaucoma[43], microcephaly[44] and others. No existing study has used handheld OCT in idiopathic intracranial hypertension (IIH), craniosynostosis or space occupying lesion. Advantages of using handheld OCT in children at risk of IH include the following:

- Handheld OCT can be performed in awake babies and infants, without the need for general anaesthesia;
- Even if under general anaesthesia, handheld OCT can be performed in a child lying down, both in the supine and lateral decubitus positions;
- Handheld OCT scans can be easily repeated in the clinical setting, enabling the assessment of evolving changes over time;
- Handheld OCT semi-automated segementation parameters can be fully controlled by the investigator.[36, 37]

For peer review only

Objectives

Primary objective

To assess the diagnostic accuracy of OCT for paediatric IH.

Secondary objectives

- To assess success rates of HH-OCT in children with craniosynostosis ± other children at risk of IH;
- To evaluate ophthalmological outcomes, including visual acuity, in this cohort and to compare these with corresponding OCT data;
- To characterise optic nerve head and retinal morphology in conditions associated with paediatric IH;
- To compare repeated OCT data between clinic visits ± ICP assessments ± cranial vault expansion surgery ± follow-up, with ICP assessments wherever possible;

For peer review only - http://bmjopen.bmj.com/site/about/guidelines.xhtml

RIO Study Protocol

- To compare OCT data in children at risk of IH with that of healthy age-matched controls from our ongoing paediatric HH-OCT normative database.[36, 37]

For peer review only

For peer review only - http://bmjopen.bmj.com/site/about/guidelines.xhtml

Methods

Study design

Single-centre, prospective diagnostic accuracy study.

Subjects

Children aged 0-18 who are diagnosed with conditions associated with IH, including craniosynostosis of all forms (syndromic and non-syndromic), IIH, space occupying lesion and others.

Recruitment

Subjects will be recruited from the ophthalmology clinic at Great Ormond Street Hospital (GOSH), London. In addition, subjects will be recruited from the admissions ward prior to 48-hour ICP assessment and/or cranial vault expansion surgery, should this be their first stage of clinical care when referred to GOSH.

Inclusion criteria

- Patients aged 0-18;
- Clinical and/or genetic diagnosis of craniosynostosis, IIH, space occupying lesion or other conditions association with IH.

Exclusion criteria

- Patients older than 18 years of age;
- Patients not wishing to participate;
- Patients incapable of giving consent and without a legal guardian willing or able to do so.

Information and consent process

Patients presenting to their pre-existing clinical appointments at GOSH, along with their family members/guardians, will be approached by the study investigator(s) and will be provided with the study information leaflet. Information leaflets suitable for children are also available. If children over 7 years old are able to understand the study, they will be given the option to provide their signature as a form of assent on the consent form along with their parent/guardian’s signature.

After the consent form is signed, patients will be taken to the investigator’s clinic room, or the handheld OCT scanner will be brought to them if appropriate, in order to acquire their OCT images. If it is not possible to take their scan on the same day, patients will be invited back to have their OCT scan at a suitable date in the near future. Where possible, subjects will have repeat OCT scans in future appointments to obtain longitudinal data. Consent will be taken in advance of ICP assessment or any surgical procedure requiring general anaesthesia.

Outcome measures

Our primary outcome measures are OCT examinations and ICP assessments. OCT examination must be performed at the time of ICP assessment, or within the 3 months prior to or 14 days following ICP assessment. Only one visit per child for OCT examination is required to fulfil the primary objective.

Our secondary outcome measures include the following, where possible:

- Success rates of HH-OCT in children at risk of IH, particularly in craniosynostosis;

For peer review only - http://bmjopen.bmj.com/site/about/guidelines.xhtml

- Repeated OCT data between clinic visits ± ICP assessments ± cranial vault expansion surgery ± follow-up, with repeated ICP data if available;
- Fundoscopic examination within 3 months of OCT examination and/or at most recent follow-up visit;
- Visual acuity (VA) measured within three months of OCT examination and/or at most recent follow-up visit;
- Contrast sensitivity measured within three months of OCT examination and/or at most recent follow-up visit;
- Visual field testing within three months of OCT examination and/or at most recent follow-up visit;
- Visual evoked potentials (VEPs) within three months of OCT examination and/or at most recent follow-up visit.
- Comparison of the above measures with ICP and final VA, where available and appropriate.

Handheld OCT image acquisition and analysis

A noncontact, spectral domain HH-OCT scanner (ENVISU C class 2300; Leica Microsystems) will be used to scan the optic nerve head (ONH) and fovea. A 12×8-mm scanning window will be used in the acquisition protocol. The 3-dimensional raster scan for both scan sequences will consist of 80 B-scans and 600 A-scans per B-scan line resulting in a short acquisition time (1.9 seconds) enabling imaging of the ONH and fovea with minimal movement artefact. HH-OCT will be performed during initial and all follow-up appointments until 48-hour ICP monitoring ± surgery ± follow-up appointments where applicable. Up to two images of the ONH and fovea will be acquired per patient per appointment.

OCT data analysis will be performed using ImageJ (https://imagej.nih.gov/ij/). Optic nerve and retinal analyses will be based on single B-scans through the deepest part of the optic cup and foveal pit, respectively. Nasal and temporal ppRNFL thickness are included in the primary aim, whilst others are exploratory.

Cup and disc parameters will include the following:

- Cup depth
- Cup width
- Cup area
- Disc width

Lateral measurements will be expressed as visual angles rather than distances since disc and cup diameters remain relatively constant in the developing eye or with changing axial length.[36]

Rim parameters will include the following:

- Rim width
- Rim height (nasal/temporal)
- Rim area
- Nasal and temporal ppRNFL thickness
- Total retinal thickness
- Bruch's membrane opening-minimum rim width (BMO-MRW, nasal/temporal)
- Bruch's membrane orientation
- Full peripapillary analysis can be performed wherever possible

For peer review only - http://bmjopen.bmj.com/site/about/guidelines.xhtml

Retinal parameters will include the following:

- Macular and perimacular retinal thickness
- Foveal pit width
- Foveal pit depth
- Foveal pit area
- Segmentation of all retinal layers.

ICP assessment

As per standard clinical care at GOSH, patients are admitted for gold-standard, 48-hour ICP assessments. An intraparenchymal catheter and bolt system is used (RAUMEDIC Neurovent-P, RAUMEDIC AG, Helmbrechts, Germany). The neurosurgeon measuring ICP will be masked to the OCT findings. Due to lack of agreement over what ICP values constitute IH in children, two definitions will be used for IH:

Definition 1: Grand mean ICP ≥15 mmHg, or ICP traces with ≥2 ICP spikes at least double the baseline ICP and ≥15mmHg for ≥5 minutes.

Definition 2: Grand mean ICP ≥20 mmHg, or ICP traces with ≥2 ICP spikes at least double the baseline ICP and ≥20 mmHg for ≥5 minutes.

ICP assessments using both definitions will be included in multi-level statistical analyses.

Secondary outcome data assessment

Fundoscopic examination of the optic discs will be performed by experienced paediatric ophthalmologists and will be graded as follows: normal, swollen, pale, atrophic. Chart logMAR VA will be measured wherever possible using the Thomson Test Chart (Thomson Software Solutions, Hatfield, UK), which uses crowded letter optotypes. If the child is unable to take part in the Thomson Test Chart, then the Keeler logMAR picture test will be used (Keeler Ltd, Windsor, UK). This test requires the child to match easily recognisable pictures (e.g. house, car, apple) and is indicated in verbal children who cannot match letter optotypes.[45] In preverbal children, we will use Teller Acuity Cards (Precision Vision, Woodstock, IL, USA) for preferential looking testing. Contrast sensitivity will be measured where possible using the Thomson Test Chart contrast sensitivity module, similar to the Pelli-Robson test (Thomson Software Solutions, Hatfield, UK). Visual field testing will be performed where possible using the Octopus 900 (Haag Streit UK Ltd, Harlow, UK) and/or Humphrey Field Analyzer 3 (Carl Zeiss Ltd, Cambridge, UK). Wherever possible, VEP assessments will be performed and graded according to a modified criterion created by Thompson *et al*.[46]

Sample size calculation

A cohort of at least 39 children is required to demonstrate at least 40% improvement in sensitivity compared to 22% demonstrated by fundoscopy demonstrated by Tuite *et al*[27] (McNemar's Z-test: n=39; power=80%, α=0.05). This would demonstrate sensitivity of at least 62%, a conservative estimate comparable to the least sensitive OCT measure demonstrated by Swanson *et al*[16] (maximal retinal thickness, 63% sensitivity) for detecting IH in children with craniosynostosis.

Statistical analysis

Diagnostic accuracy testing will be performed to calculate sensitivity, specificity, positive predictive value, negative predictive value and accuracy of OCT parameters to detect IH, using the OCT

For peer review only - http://bmjopen.bmj.com/site/about/guidelines.xhtml

parameters and two definitions of IH described above. Receiver operating characteristic (ROC) curves will be plotted to evaluate diagnostic accuracy of OCT parameters for IH. Mixed model regression analysis will be performed to assess the strength of association between OCT parameters and secondary outcome measures, described above.

Timing

Our study commenced in February 2020 but was halted in between March 2020 and September 2020 due to COVID-19 restrictions in the United Kingdom. Our study restarted in September 2020, therefore at the time of writing, data collection is still at an early stage. We aim to finalise this study by February 2024.

Assessment of possible adverse effects

OCT is a safe and non-contact imaging technique that uses low-coherence light. It is extremely unlikely that any adverse events could arise during OCT imaging. Standard clinical care will continue during and beyond the completion of the RIO Study.

Patient and public involvement statement

Patients, families and the public will be actively involved in the RIO Study via two advisory groups: i) the Young Person's Advisory Group (YPAG), including participants from the GOSH YPAG and Moorfields Eye-YPAG; ii) the GOSH Parent and Carer Advisory Group (PCAG). Participants in both advisory groups will assist the RIO investigators in several ways, including but not limited to: i) sharing their lived experience of the conditions being studied; ii) keeping the study child-friendly and parent-friendly; iii) keeping recruitment rates high; iv) dissemination of study findings. Both advisory groups will meet at least annually.

Ethics and dissemination

Ethical approval was granted for this study by the East Midlands Nottingham 2 Research Ethics committee (UOL0348/IRAS 105137). Participants will already be under the care of GOSH, with continuity of care beyond our study. Our research clinic will involve rapid, non-invasive OCT imaging during the patients' existing appointments ± additional appointments only where required. Our study will not affect the overall management of the children. Consent will be sought from parents/guardians. Our findings will be disseminated via relevant local and national/international meetings, as well as via peer reviewed journal publication and social media.

For peer review only

For peer review only - http://bmjopen.bmj.com/site/about/guidelines.xhtml

Authors' contributions

SRR: Concept, protocol writing, statistical methods, final approval.

NUOJ: Concept, protocol writing, critical revision, final approval.

RB: Concept, protocol writing, critical revision, final approval.

CB: Concept, protocol writing, statistical methods, critical revision, final approval.

FAP: Concept, protocol writing, statistical methods, critical revision, final approval.

IG: Concept, protocol writing, critical revision, statistical methods, overall supervision, final approval.

For peer review only

Acknowledgements

We would like to thank the participants of GOSH Young Persons Advisory Group (YPAG), Moorfields Eye YPAG and GOSH Parent and Carer Advisory Group for supporting this study. This work is supported by the Medical Research Council, UK (Grant No. MR/N004566/1). Dr Sohaib Rufai is funded by the National Institute of Health Research Doctoral Fellowship Award. This work is supported by the NIHR Great Ormond Street Hospital Biomedical Research Centre. This protocol proposes independent research funded by the National Institute for Health Research (NIHR) and the Medical Research Council, UK. The views expressed are those of the author(s) and not necessarily those of the MRC, the NHS, the NIHR or the Department of Health and Social Care.

For peer review only - http://bmjopen.bmj.com/site/about/guidelines.xhtml

RIO Study Protocol

References

1. Quincke, H. Ueber Meningitis serosa und verwandte Zustände. *Deutsche Zeitschrift f. Nervenheilkunde* **9,** 149–168 (1896). https://doi.org/10.1007/BF01668270
2. Derderian C, Seaward J. Syndromic craniosynostosis. Semin Plast Surg. 2012 May;26(2):64-75. doi: 10.1055/s-0032-1320064. PMID: 23633933; PMCID: PMC3424695.
3. Jordan CO, Aylward SC. Intracranial hypertension: a current review. Curr Opin Pediatr. 2018 Dec;30(6):764-774. doi: 10.1097/MOP.0000000000000689. PMID: 30188410.
4. Aylward SC, Reem RE. Pediatric Intracranial Hypertension. Pediatr Neurol. 2017 Jan;66:32-43. doi: 10.1016/j.pediatrneurol.2016.08.010. Epub 2016 Sep 17. PMID: 27940011.
5. Matthews YY, Dean F, Lim MJ, Mclachlan K, Rigby AS, et al. Pseudotumor cerebri syndrome in childhood: incidence, clinical profile and risk factors in a national prospective population-based cohort study. Arch Dis Child. 2017 Aug;102(8):715-721. doi: 10.1136/archdischild-2016-312238. Epub 2017 Mar 29. PMID: 28356250.
6. Gillson N, Jones C, Reem RE, Rogers DL, Zumberge N, Aylward SC. Incidence and Demographics of Pediatric Intracranial Hypertension. Pediatr Neurol. 2017 Aug;73:42-47. doi: 10.1016/j.pediatrneurol.2017.04.021. Epub 2017 Apr 27. PMID: 28668233.
7. Mollan SP, Davies B, Silver NC, Shaw S, Mallucci CL, et al. Idiopathic intracranial hypertension: consensus guidelines on management. J Neurol Neurosurg Psychiatry. 2018 Oct;89(10):1088-1100. doi: 10.1136/jnnp-2017-317440. Epub 2018 Jun 14. PMID: 29903905; PMCID: PMC6166610.
8. Radhakrishnan K, Thacker AK, Bohlaga NH, Maloo JC, Gerryo SE. Epidemiology of idiopathic intracranial hypertension: a prospective and case-control study. J Neurol Sci. 1993 May;116(1):18-28. doi: 10.1016/0022-510x(93)90084-c. PMID: 8509801.
9. Raoof N, Sharrack B, Pepper IM, Hickman SJ. The incidence and prevalence of idiopathic intracranial hypertension in Sheffield, UK. Eur J Neurol. 2011 Oct;18(10):1266-8. doi: 10.1111/j.1468-1331.2011.03372.x. Epub 2011 Mar 22. PMID: 21426442.
10. Radhakrishnan K, Ahlskog JE, Cross SA, Kurland LT, O'Fallon WM. Idiopathic intracranial hypertension (pseudotumor cerebri). Descriptive epidemiology in Rochester, Minn, 1976 to 1990. Arch Neurol. 1993 Jan;50(1):78-80. doi: 10.1001/archneur.1993.00540010072020. PMID: 8418804.
11. Cinciripini GS, Donahue S, Borchert MS. Idiopathic intracranial hypertension in prepubertal pediatric patients: characteristics, treatment, and outcome. Am J Ophthalmol. 1999 Feb;127(2):178-82. doi: 10.1016/s0002-9394(98)00386-9. PMID: 10030560.
12. Kesler A, Fattal-Valevski A. Idiopathic intracranial hypertension in the pediatric population. J Child Neurol. 2002 Oct;17(10):745-8. doi: 10.1177/08830738020170101401. PMID: 12546428.
13. Babikian P, Corbett J, Bell W. Idiopathic intracranial hypertension in children: the Iowa experience. J Child Neurol. 1994 Apr;9(2):144-9. doi: 10.1177/088307389400900208. PMID: 8006364.
14. Phillips PH, Repka MX, Lambert SR. Pseudotumor cerebri in children. J AAPOS. 1998 Feb;2(1):33-8. doi: 10.1016/s1091-8531(98)90107-0. PMID: 10532364.
15. Tamburrini G, Caldarelli M, Massimi L, Santini P, Di Rocco C. Intracranial pressure monitoring in children with single suture and complex craniosynostosis: a review. Childs Nerv Syst. 2005 Oct;21(10):913-21. doi: 10.1007/s00381-004-1117-x. Epub 2005 May 3. PMID: 15871027.
16. Swanson JW, Aleman TS, Xu W, Ying GS, Pan W, et al Evaluation of Optical Coherence Tomography to Detect Elevated Intracranial Pressure in Children. JAMA Ophthalmol. 2017 Apr 1;135(4):320-328. doi: 10.1001/jamaophthalmol.2017.0025. PMID: 28241164; PMCID: PMC5470406.
17. Renier D, Sainte-Rose C, Marchac D, Hirsch JF. Intracranial pressure in craniostenosis. J Neurosurg. 1982 Sep;57(3):370-7. doi: 10.3171/jns.1982.57.3.0370. PMID: 7097333.

For peer review only - http://bmjopen.bmj.com/site/about/guidelines.xhtml

18. Wall SA, Thomas GP, Johnson D, Byren JC, Jayamohan J, et al. The preoperative incidence of raised intracranial pressure in nonsyndromic sagittal craniosynostosis is underestimated in the literature. J Neurosurg Pediatr. 2014 Dec;14(6):674-81. doi: 10.3171/2014.8.PEDS1425. Epub 2014 Sep 26. PMID: 25259602.
19. Gault DT, Renier D, Marchac D, Jones BM. Intracranial pressure and intracranial volume in children with craniosynostosis. Plast Reconstr Surg. 1992 Sep;90(3):377-81. doi: 10.1097/00006534-199209000-00003. PMID: 1513883.
20. Arnaud E, Renier D, Marchac D. Prognosis for mental function in scaphocephaly. J Neurosurg. 1995 Sep;83(3):476-9. doi: 10.3171/jns.1995.83.3.0476. PMID: 7666225.
21. Eide PK, Helseth E, Due-Tønnessen B, Lundar T. Assessment of continuous intracranial pressure recordings in childhood craniosynostosis. Pediatr Neurosurg. 2002 Dec;37(6):310-20. doi: 10.1159/000066311. PMID: 12422046.
22. Pople IK, Muhlbauer MS, Sanford RA, Kirk E. Results and complications of intracranial pressure monitoring in 303 children. Pediatr Neurosurg. 1995;23(2):64-7. doi: 10.1159/000120938. PMID: 8555097.
23. Gambardella G, Zaccone C, Cardia E, Tomasello F. Intracranial pressure monitoring in children: comparison of external ventricular device with the fiberoptic system. Childs Nerv Syst. 1993 Dec;9(8):470-3. doi: 10.1007/BF00393552. PMID: 8124675.
24. Eide PK, Helseth E, Due-Tønnessen B, Lundar T. Changes in intracranial pressure after calvarial expansion surgery in children with slit ventricle syndrome. Pediatr Neurosurg. 2001 Oct;35(4):195-204. doi: 10.1159/000050421. PMID: 11694797.
25. Hayward R, Britto J, Dunaway D, Jeelani O. Connecting raised intracranial pressure and cognitive delay in craniosynostosis: many assumptions, little evidence. J Neurosurg Pediatr. 2016 Aug;18(2):242-50. doi: 10.3171/2015.6.PEDS15144. Epub 2016 May 13. PMID: 27176895.
26. Czosnyka M, Pickard JD. Monitoring and interpretation of intracranial pressure. J Neurol Neurosurg Psychiatry. 2004 Jun;75(6):813-21. doi: 10.1136/jnnp.2003.033126. PMID: 15145991; PMCID: PMC1739058.
27. Tuite GF, Chong WK, Evanson J, Narita A, Taylor D, et al. The effectiveness of papilledema as an indicator of raised intracranial pressure in children with craniosynostosis. Neurosurgery. 1996 Feb;38(2):272-8. doi: 10.1097/00006123-199602000-00009. PMID: 8869054.
28. Tuite GF, Evanson J, Chong WK, Thompson DN, Harkness WF, Jones BM, Hayward RD. The beaten copper cranium: a correlation between intracranial pressure, cranial radiographs, and computed tomographic scans in children with craniosynostosis. Neurosurgery. 1996 Oct;39(4):691-9. doi: 10.1097/00006123-199610000-00007. PMID: 8880760.
29. Driessen C, Bannink N, Lequin M, van Veelen ML, Naus NC, et al. Are ultrasonography measurements of optic nerve sheath diameter an alternative to funduscopy in children with syndromic craniosynostosis? J Neurosurg Pediatr. 2011 Sep;8(3):329-34. doi: 10.3171/2011.6.PEDS10547. PMID: 21882928.
30. Lee H, Proudlock FA, Gottlob I. Pediatric Optical Coherence Tomography in Clinical Practice-Recent Progress. Invest Ophthalmol Vis Sci. 2016 Jul 1;57(9):OCT69-79. doi: 10.1167/iovs.15-18825. PMID: 27409508.
31. Chen CL, Bojikian KD, Xin C, Wen JC, Gupta D, et al. Repeatability and reproducibility of optic nerve head perfusion measurements using optical coherence tomography angiography. J Biomed Opt. 2016 Jun 1;21(6):65002. doi: 10.1117/1.JBO.21.6.065002. PMID: 27272095; PMCID: PMC4896902.
32. Rufai SR, Jeelani NUO, McLean RJ. Early Recognition of Raised Intracranial Pressure in Craniosynostosis Using Optical Coherence Tomography. J Craniofac Surg. 2021 Jan-Feb 01;32(1):201-205. doi: 10.1097/SCS.0000000000006771. PMID: 33185414; PMCID: PMC7769183.

For peer review only - http://bmjopen.bmj.com/site/about/guidelines.xhtml

RIO Study Protocol

33. Dagi LR, Tiedemann LM, Heidary G, Robson CD, Hall AM, Zurakowski D. Using spectral-domain optical coherence tomography to detect optic neuropathy in patients with craniosynostosis. J AAPOS. 2014 Dec;18(6):543-9. doi: 10.1016/j.jaapos.2014.07.177. PMID: 25498464.
34. Driessen C, Eveleens J, Bleyen I, van Veelen ML, Joosten K, Mathijssen I. Optical coherence tomography: a quantitative tool to screen for papilledema in craniosynostosis. Childs Nerv Syst. 2014 Jun;30(6):1067-73. doi: 10.1007/s00381-014-2376-9. Epub 2014 Feb 12. PMID: 24519451.
35. Lee H, Proudlock F, Gottlob I. Is handheld optical coherence tomography reliable in infants and young children with and without nystagmus? Invest Ophthalmol Vis Sci. 2013 Dec 17;54(13):8152-9. doi: 10.1167/iovs.13-13230. PMID: 24222299; PMCID: PMC4054919.
36. Patel A, Purohit R, Lee H, Sheth V, Maconachie G, et al. Optic Nerve Head Development in Healthy Infants and Children Using Handheld Spectral-Domain Optical Coherence Tomography. Ophthalmology. 2016 Oct;123(10):2147-57. doi: 10.1016/j.ophtha.2016.06.057. Epub 2016 Aug 9. PMID: 27521172; PMCID: PMC5036922.
37. Lee H, Purohit R, Patel A, Papageorgiou E, Sheth V, et al. In Vivo Foveal Development Using Optical Coherence Tomography. Invest Ophthalmol Vis Sci. 2015 Jul;56(8):4537-45. doi: 10.1167/iovs.15-16542. PMID: 26200492.
38. Anwar S, Nath M, Patel A, Lee H, Brown S, et al. Potential utility of fovael morphology in preterm infants measured using hand-held optical coherence tomography in retinopathy of prematurity screening. Retina. 2020 Aug;40(8):1592-1602. doi: 10.1097/IAE.0000000000002622. PMID: 31356497; PMCID: PMC7392578.
39. Rufai SR, Thomas MG, Purohit R, Bunce C, Lee H, et al. Can Structural Grading of Foveal Hypoplasia Predict Future Vision in Infantile Nystagmus?: A Longitudinal Study. Ophthalmology. 2020 Apr;127(4):492-500. doi: 10.1016/j.ophtha.2019.10.037. Epub 2019 Nov 4. PMID: 31937464; PMCID: PMC7105819.
40. Pilat A, Sibley D, McLean RJ, Proudlock FA, Gottlob I. High-Resolution Imaging of the Optic Nerve and Retina in Optic Nerve Hypoplasia. Ophthalmology. 2015 Jul;122(7):1330-9. doi: 10.1016/j.ophtha.2015.03.020. Epub 2015 May 1. PMID: 25939636; PMCID: PMC4518044.
41. Thomas MG, Kumar A, Kohl S, Proudlock FA, Gottlob I. High-resolution in vivo imaging in achromatopsia. Ophthalmology. 2011 May;118(5):882-7. doi: 10.1016/j.ophtha.2010.08.053. Epub 2011 Jan 6. PMID: 21211844.
42. Mohammad S, Gottlob I, Sheth V, Pilat A, Lee H, et al. Characterization of Abnormal Optic Nerve Head Morphology in Albinism Using Optical Coherence Tomography. Invest Ophthalmol Vis Sci. 2015 Jul;56(8):4611-8. doi: 10.1167/iovs.15-16856. PMID: 26200501.
43. Pilat AV, Proudlock FA, Shah S, Sheth V, Purohit R, et al. Assessment of the anterior segment of patients with primary congenital glaucoma using handheld optical coherence tomography. Eye (Lond). 2019 Aug;33(8):1232-1239. doi: 10.1038/s41433-019-0369-3. Epub 2019 Mar 18. PMID: 30886322; PMCID: PMC7005739.
44. Papageorgiou E, Pilat A, Proudlock F, Lee H, Purohit R, et al. Retinal and optic nerve changes in microcephaly: An optical coherence tomography study. Neurology. 2018 Aug 7;91(6):e571-e585. doi: 10.1212/WNL.0000000000005950. Epub 2018 Jul 11. PMID: 29997194; PMCID: PMC6105049.
45. O'Boyle C, Chen SI, Little JA. Crowded letter and crowded picture logMAR acuity in children with amblyopia: a quantitative comparison. Br J Ophthalmol. 2017 Apr;101(4):457-461. doi: 10.1136/bjophthalmol-2015-307677. Epub 2016 Jul 7. PMID: 27388249; PMCID: PMC5583677.
46. Thompson DA, Liasis A, Hardy S, Hagan R, Hayward RD, et al. Prevalence of abnormal pattern reversal visual evoked potentials in craniosynostosis. Plast Reconstr Surg. 2006 Jul;118(1):184-92. doi: 10.1097/01.prs.0000220873.72953.3e. PMID: 16816694.

For peer review only - http://bmjopen.bmj.com/site/about/guidelines.xhtml

Recognition of Intracranial Hypertension using Handheld Optical Coherence Tomography in Children (The RIO Study): A Diagnostic Accuracy Study Protocol

Journal:	*BMJ Open*
Manuscript ID	bmjopen-2021-048745.R1
Article Type:	Protocol
Date Submitted by the Author:	26-Aug-2021
Complete List of Authors:	Rufai, Sohaib; Great Ormond Street Hospital For Children NHS Foundation Trust, Clinical and Academic Department of Ophthalmology; University of Leicester, University of Leicester Ulverscroft Eye Unit<br>Jeelani, Noor ul Owase; Great Ormond Street Hospital For Children NHS Foundation Trust, Craniofacial Unit; UCL, Institute of Child Health<br>Bowman, Richard; Great Ormond Street Hospital for Children NHS Foundation Trust, Clinical and Academic Department of Ophthalmology<br>Bunce, Catey; Royal Marsden Hospital NHS Trust, Clinical Trials Unit<br>Proudlock, Frank; University of Leicester, University of Leicester Ulverscroft Eye Unit<br>Gottlob, Irene; University of Leicester, University of Leicester Ulverscroft Eye Unit
**Primary Subject Heading**:	Ophthalmology
Secondary Subject Heading:	Diagnostics, Neurology, Paediatrics, Surgery, Evidence based practice
Keywords:	OPHTHALMOLOGY, Paediatric neurosurgery < PAEDIATRIC SURGERY, Paediatric ophthalmology < OPHTHALMOLOGY, Paediatric plastic & reconstructive surgery < PAEDIATRIC SURGERY, Neuro-ophthalmology < NEUROLOGY, Paediatric neurology < PAEDIATRICS

RIO Study Protocol

Title Page

Title: Recognition of Intracranial Hypertension using Handheld Optical Coherence Tomography in Children (The RIO Study): A Diagnostic Accuracy Study Protocol

Short title: The RIO Study Protocol

Authors: Sohaib R. Rufai (BMBS, MRes)[1-3], Noor ul Owase Jeelani (MPhil, FRCS)[2], Richard Bowman (MD, FRCOphth)[1], Catey Bunce (MSc, DSc)[4], Frank A. Proudlock (MSc, PhD)[3], Irene Gottlob (MD, FRCOphth)[3]

Affiliations:

1. Clinical and Academic Department of Ophthalmology, Great Ormond Street Hospital for Children NHS Foundation Trust and UCL Great Ormond Street Institute of Child Health, London, United Kingdom.
2. Craniofacial Unit, Great Ormond Street Hospital for Children NHS Foundation Trust and UCL Great Ormond Street Institute of Child Health, London, United Kingdom.
3. The University of Leicester Ulverscroft Eye Unit, Leicester Royal Infirmary, Leicester, United Kingdom.
4. Clinical Trials Unit, The Royal Marsden NHS Trust, London, United Kingdom.

Corresponding author: Dr Sohaib R. Rufai; E-mail: Sohaib.Rufai@nhs.net

Sponsor: University of Leicester

Sponsor Reference Number: 0348

Ethics Reference: 12/EM0261

Protocol Date: 01/11/2019

Competing Interests Statement: Dr. Proudlock reports personal fees from Leica Microsystems to run a virtual clinical symposium for hand-held optical coherence tomography, outside the submitted work. Dr. Rufai, Dr. Jeelani, Dr. Bowman, Dr. Bunce and Prof. Gottlob have no competing interests to report.

Data Availability Statement: No data are available as this is a study protocol.

Funding Statement: This work is supported by the Medical Research Council, UK (Grant No. MR/N004566/1). Dr Sohaib Rufai is funded by the National Institute of Health Research Doctoral Fellowship (Award ID: NIHR300155). This work is supported by the NIHR Great Ormond Street Hospital Biomedical Research Centre. This protocol proposes independent research funded by the National Institute for Health Research (NIHR) and the Medical Research Council, UK. The views expressed are those of the author(s) and not necessarily those of the MRC, the NHS, the NIHR or the Department of Health and Social Care.

For peer review only - http://bmjopen.bmj.com/site/about/guidelines.xhtml

For peer review only

RIO Study Protocol

Abstract

Introduction

Paediatric intracranial hypertension (IH) is a rare but serious condition that can pose deleterious effects on the brain and vision. Estimating intracranial pressure (ICP) in children is difficult. Gold standard direct ICP measurement is invasive and carries risk. It is impractical to routinely perform direct ICP measurements over time for all children at risk of IH. This study proposes to assess the diagnostic accuracy of handheld optical coherence tomography (handheld OCT), a non-invasive ocular imaging method, to detect IH in children.

Methods and analysis

This is a single-centre prospective study evaluating the diagnostic accuracy of handheld OCT for IH in at risk children. Inclusion criteria include clinical and/or genetic diagnosis of craniosynostosis, idiopathic intracranial hypertension (IIH), space occupying lesion or other conditions association with IH and age 0-18 years old. Exclusion criteria include patients older than 18 years of age and/or absence of condition placing the child at risk of IH. The primary outcome measures are handheld OCT and 48-hour ICP assessments, which will be used for diagnostic accuracy testing (sensitivity, specificity, positive predictive value, negative predictive value and accuracy). Main secondary outcome measures include visual acuity, fundoscopic examination, contrast sensitivity, visual field testing and visual evoked potentials, wherever possible.

Ethics and dissemination

Ethical approval was granted for this study by the East Midlands Nottingham 2 Research Ethics committee (UOL0348/IRAS 105137). Our findings will be disseminated through presentation at relevant meetings, peer-reviewed publication and via the popular media.

Trial registration number: ISRCTN52858719

Strengths and limitations of this study

- This the first diagnostic accuracy study protocol using handheld optical coherence tomography (OCT) to detect intracranial hypertension (IH) in at risk children.
- This protocol promotes transparency of our methodology and reduces the risk of duplication.
- Fully automated handheld OCT image analysis is not currently available and manual segmentation takes longer, albeit our evidence-based methodology features semi-automated segmentation.
- Although handheld OCT can provide *in vivo* imaging in infants without sedation, it could be especially challenging in this patient population, which could limit imaging success rates.
- Gold standard intracranial pressure measurements form our reference standard, but these are only performed in select patients, thus limiting our overall recruitment.

For peer review only - http://bmjopen.bmj.com/site/about/guidelines.xhtml

For peer review only

RIO Study Protocol

Introduction

Intracranial hypertension

First described by Quincke in 1896, intracranial hypertension (IH) remains an area of major importance among the medical profession.[1] If undetected and untreated, IH can pose devastating consequences including visual impairment, cognitive impairment, disability and even death.[2, 3] This is of particular significance in subacute conditions affecting children, because insidious elevation of intracranial pressure (ICP) may only be detected after insult to the brain and vision has already begun.[2, 4] Therefore, it is crucial that IH is detected and treated as early as possible to prevent or limit the sequelae of IH.

The incidence of IH is approximately 0.63-0.71 per 100,000 children.[5, 6] IH can be categorised as primary, also known as idiopathic intracranial hypertension (IIH), and secondary; the former is characterised by elevated ICP with no identifiable cause, while the latter is characterised by elevated ICP directly resulting from another condition.[4, 7] Common associations of primary IH include female sex, high body mass index (BMI) and postpubertal status, however it can occur in children with no predilection for sex or high BMI.[4, 8-14] Examples of secondary IH include expansive skull pathology, i.e. space occupying lesion or hydrocephalus, or constricting skull pathology, such as craniosynostosis, characterised by premature fusion of cranial sutures.[2-4]

Measuring intracranial pressure

Measuring ICP in children presents a number of challenges. The gold standard method is direct intraparenchymal measurement, but this involves hospital admission and carries significant risks including infection, bleeding, CSF leak, mechanical failure and general anaesthesia risks.[15] Another limitation of intraparenchymal measurement is the difficulty in obtaining repeated measures over time. Furthermore, there is no clinical consensus on timing, frequency and duration for accurate measurement, or what figure constitutes raised ICP in children. Many consider ICP greater than 15mmHg to be elevated[2, 16-24], but Hayward *et al*[25] argue this could reasonably be raised to 20 mmHg.[25] Overnight ICP monitoring during natural sleep, which provides a ‘‘grand average’’ with description of the dynamics of the ICP, is considered the ‘‘gold standard’’.[26] In Great Ormond Street Hospital for Children (GOSH), London, monitoring is performed using an ICP bolt (Raumedic AG, Helmbrechts, Germany) for 48 hours according to clinical discretion, particularly in children with clinical suspicion of IH where ophthalmological findings, including fundoscopic and electrodiagnostic findings, are equivocal. This represents an important patient cohort where a more sensitive non-invasive measure could improve clinical decision making and reduce the need for ICP bolt monitoring. However, the youngest in this group undergoing ICP bolt monitoring are typically toddlers and older, whereas those aged under 1 year with conditions such as craniosynostosis typically undergo surgical treatment expectantly rather than having ICP bolt monitoring.

The ideal measure of ICP would be non-invasive, acceptable for children, quantitative and would permit serial measurements to appreciate evolving changes related to rising ICP. Existing surrogate measures of ICP do not fulfil all these criteria, including fundoscopy[27], radiography[28] and ocular ultrasonography[29], each failing to provide adequate sensitivity to be used as screening tools. In particular, fundoscopy is routinely used in paediatric ophthalmology clinics to detect IH. Tuite *et al*[27] demonstrated that fundoscopy yielded sensitivity of only 22% for IH in children with craniosynostosis aged under 8 years old, while specificity was 98%. Hence, papilloedema detected

For peer review only - http://bmjopen.bmj.com/site/about/guidelines.xhtml

on fundoscopy reliably indicates IH in younger children with craniosynostosis, but crucially its absence does not exclude IH.

Optical coherence tomography

The optic nerve is primarily intracranial, hence IH can cause optic nerve swelling with secondary retinal changes. Optical coherence tomography (OCT) is a non-invasive imaging modality that can rapidly produce ultra-high resolution cross-sectional images of the optic nerve and retina *in vivo*.[30] OCT scans of the optic nerve head can provide highly reproducible quantitative measures associated with IH within seconds.[31] Adult studies have demonstrated the value of OCT in IH, including volumetric analysis.[32-34]

Our recent systematic review[35] revealed twenty-one studies using OCT to assist diagnosis and management of IH in children with craniosynostosis, IIH, space occupying lesion and other pathology. This review revealed generally good diagnostic capability for OCT, but no level 1 evidence was identified. There was inconsistency in OCT parameters and reference standards used, with no study featuring overnight ICP monitoring. Validating prospective research is therefore required to determine optimal OCT parameters in this role and to develop formal clinical guidelines. Moreover, no studies used handheld OCT in children with craniosynostosis, IIH or space occupying lesion.

Handheld optical coherence tomography

OCT has revolutionised the clinical practice of ophthalmology over the past two decades. However, infants have previously been largely deprived of this technology as conventional OCT machines were designed for adults, requiring compliance with a chin rest, good fixation and cooperation. These limitations have been overcome with the recent advent of the handheld spectral-domain OCT designed for infants, which is demonstrably reliable in awake infants.[30, 36] Normal development of the optic nerve[37] and retina[38] in children has recently been described using handheld OCT. A wide range of conditions have also been investigated using handheld OCT in children, including retinopathy of prematurity[39], nystagmus[40], foveal hypoplasia[41], optic nerve hypoplasia[41], achromatopsia[42], albinism[43], primary congenital glaucoma[44], microcephaly[45], craniopagus twins[46] and others.

More recently, we performed a feasibility and repeatability study[47] using handheld OCT in children with craniosynostosis (n=50, median age 51 months, age range 2–157 months). We found good feasibility, with 86% achieving at least unilateral OCT and 76% achieving bilateral OCTs of the optic nerve head (ONH). This was higher than the success rate in healthy children found by Patel et al[37] (70% achieving at least unilateral OCTs of the ONH). Factors boosting the likelihood of success in children with craniosynostosis included good understanding and cooperation of the child and parent/guardian and availability of an assistant. We also performed repeatability analysis in 20 children and found good repeatability (intraclass correlation coefficient [ICC] range, 0.77–0.99; the majority exceeded 0.90).

Advantages of using handheld OCT in children at risk of IH include the following:

- Handheld OCT can be performed in awake babies and infants, without the need for general anaesthesia;
- Even if under general anaesthesia, handheld OCT can be performed in a child lying down, both in the supine and lateral decubitus positions;
- Handheld OCT scans can be easily repeated in the clinical setting, enabling the assessment of evolving changes over time;

For peer review only - http://bmjopen.bmj.com/site/about/guidelines.xhtml

RIO Study Protocol

- Handheld OCT semi-automated segmentation parameters can be fully controlled by the investigator.[37, 38, 47]

Objectives

Primary objective

To assess the diagnostic accuracy of OCT for paediatric IH.

Secondary objectives

- To assess success rates of handheld OCT in children with craniosynostosis and other children at risk of IH;
- To evaluate ophthalmological outcomes, including visual acuity, in this cohort and to compare these with corresponding OCT data;
- To characterise optic nerve head and retinal morphology in conditions associated with paediatric IH;
- To compare repeated OCT data between clinic visits ± ICP assessments ± cranial vault expansion surgery ± follow-up, with ICP assessments wherever possible;
- To compare OCT data in children at risk of IH with that of healthy age-matched controls from our ongoing paediatric handheld OCT normative database.[37, 38]

For peer review only - http://bmjopen.bmj.com/site/about/guidelines.xhtml

Methods

Study design

Single-centre, prospective diagnostic accuracy study.

Subjects

Children aged 0-18 who are diagnosed with conditions associated with IH, including craniosynostosis of all forms (syndromic and non-syndromic), IIH, space occupying lesion and others.

Recruitment

Subjects will be recruited from the ophthalmology clinic at GOSH, London. In addition, subjects will be recruited from the admissions ward prior to 48-hour ICP assessment and/or cranial vault expansion surgery, should this be their first stage of clinical care when referred to GOSH.

Inclusion criteria

- Patients aged 0-18;
- Clinical and/or genetic diagnosis of craniosynostosis, IIH, space occupying lesion or other conditions association with IH.

Exclusion criteria

- Patients older than 18 years of age;
- Patients not wishing to participate;
- Patients incapable of giving consent and without a legal guardian willing or able to do so.

Information and consent process

Patients presenting to their pre-existing clinical appointments at GOSH, along with their family members/guardians, will be approached by the study investigator(s) and will be provided with the study information leaflet. Information leaflets suitable for children are also available. If children over 7 years old are able to understand the study, they will be given the option to provide their signature as a form of assent on the consent form along with their parent/guardian's signature.

After the consent form is signed, patients will be taken to the investigator's clinic room, or the handheld OCT scanner will be brought to them if appropriate, in order to acquire their OCT images. If it is not possible to take their scan on the same day, patients will be invited back to have their OCT scan at a suitable date in the near future. Where possible, subjects will have repeat OCT scans in future appointments to obtain longitudinal data. Consent will be taken in advance of ICP assessment or any surgical procedure requiring general anaesthesia.

Outcome measures

Our primary outcome measures are OCT examinations and ICP assessments. Our primary OCT outcome measure is retinal nerve fibre layer (RNFL) thickness; other OCT parameters are listed below. OCT examination must be performed at the time of ICP assessment, or within 28 days prior to or 14 days following ICP assessment. Only one visit per child for OCT examination is required to fulfil the primary objective.

Our secondary outcome measures include the following, where possible:

For peer review only - http://bmjopen.bmj.com/site/about/guidelines.xhtml

RIO Study Protocol

- Success rates of handheld OCT in children at risk of IH, particularly in craniosynostosis;
- Repeated OCT data between clinic visits ± ICP assessments ± cranial vault expansion surgery ± follow-up, with repeated ICP data if available;
- Fundoscopic examination within 3 months of OCT examination and/or at most recent follow-up visit;
- Visual acuity (VA) measured within three months of OCT examination and/or at most recent follow-up visit;
- Contrast sensitivity measured within three months of OCT examination and/or at most recent follow-up visit;
- Visual field testing within three months of OCT examination and/or at most recent follow-up visit;
- Visual evoked potentials (VEPs) within three months of OCT examination and/or at most recent follow-up visit.
- Comparison of the above measures with ICP and final VA, where available and appropriate.

Handheld optical coherence tomography image acquisition and analysis

A noncontact, spectral domain handheld OCT scanner (Envisu C-2300, 3.3-µm axial resolution; Leica Microsystems, Wetzlar, Germany) will be used to scan the ONH and fovea. A 12×8-mm scanning window will be used in the acquisition protocol – this permits imaging of both the ONH and fovea in one scan. The 3-dimensional raster scan for both scan sequences will consist of 100 B-scans and 500 A-scans per B-scan line resulting in a short acquisition time (1.9 seconds) enabling imaging of the ONH and fovea with minimal movement artefact. Handheld OCT will be performed during 48-hour ICP monitoring to fulfil our primary objective; wherever possible, handheld OCT will also be performed during clinic visits to fulfil our secondary objectives. In awake children, visual devices including cartoons on smartphones and tablets will be used as appropriate. Where possible, an assistant can operate the controls to acquire images, otherwise the lead investigator (SRR) will acquire images alone using the foot-pedal.

Our imaging protocol involves scanning the right eye followed by the left eye. Once the image is acquired, the en face view is used to identify the ONH, which is navigated frame by frame until the central slice featuring the deepest optic cup was identified for ONH analysis. For foveal segmentation, the fovea is navigated frame by frame until the central slice featuring the deepest foveal pit is identified. Scans are repeated twice per eye where possible, and any scans of non-analysable quality are discarded. Analysable quality is defined as an ONH tomogram wherein the edges of the disc margins and the cup profile, including its lowermost point, can be clearly visualised, or a foveal tomogram wherein it is possible to identify the foveal pit in full and all foveal layers from the external limiting membrane to Bruch's membrane. In cases of papilloedema, the handheld OCT probe can be repositioned slightly forwards in the axial plane, or the reference arm can be lowered, until the entire swollen ONH is visualised without missing the disc margins. Using this technique, all cases with papilloedema were successfully imaged in our recent feasibility and repeatability study.[47]

ImageJ 1.48 (National Institutes of Health, Bethesda, MD)[48] shall be used for quantitative segmentation and the ABSnake plugin[49] shall be used identify the internal limiting membrane contour, which can be corrected manually where required. Lateral distance measurements (defined for adults on the machine) shall be corrected to account for the smaller axial lengths in children using a conversion table according to age from the data presented by Maldonado *et al*.[50] Optic nerve and retinal analyses will be based on single B-scans through the deepest part of the optic cup and foveal pit, respectively.

For peer review only - http://bmjopen.bmj.com/site/about/guidelines.xhtml

Cup and disc parameters will include the following:

- Cup depth
- Cup width
- Cup area
- Disc width

Lateral measurements will be expressed as visual angles rather than distances since disc and cup diameters remain relatively constant in the developing eye or with changing axial length.[37]

Rim parameters will include the following:

- Rim width
- Rim height (nasal/temporal)
- Rim area
- Nasal and temporal ppRNFL thickness
- Total retinal thickness
- Bruch's membrane opening-minimum rim width (BMO-MRW, nasal/temporal)
- Bruch's membrane orientation
- Full peripapillary volumetric analysis can be performed wherever possible, using a recently published protocol[51]

Where available, retinal parameters will include the following:

- Macular and perimacular retinal thickness
- Foveal pit width
- Foveal pit depth
- Foveal pit area
- Segmentation of all retinal layers.

ICP assessment

As per standard clinical care at GOSH, patients are admitted for gold-standard, 48-hour ICP assessments. An intraparenchymal catheter and bolt system is used (RAUMEDIC Neurovent-P, RAUMEDIC AG, Helmbrechts, Germany). The neurosurgeon measuring ICP will be masked to the OCT findings. Due to lack of agreement over what ICP values constitute IH in children, two definitions will be used for IH:

Definition 1: Grand mean ICP $\geq$15 mmHg, or ICP traces with $\geq$2 ICP spikes at least double the baseline ICP and $\geq$15mmHg for $\geq$5 minutes.

Definition 2: Grand mean ICP $\geq$20 mmHg, or ICP traces with $\geq$2 ICP spikes at least double the baseline ICP and $\geq$20 mmHg for $\geq$5 minutes.

ICP assessments using both definitions will be included in multi-level statistical analyses.

Secondary outcome data assessment

Fundoscopic examination of the optic discs will be performed by experienced paediatric ophthalmologists and will be graded as follows: normal, swollen, pale, atrophic. Chart logMAR VA will be measured wherever possible using the Thomson Test Chart (Thomson Software Solutions, Hatfield, UK), which uses crowded letter optotypes. If the child is unable to take part in the Thomson Test Chart, then the Keeler logMAR picture test will be used (Keeler Ltd, Windsor, UK). This test

For peer review only - http://bmjopen.bmj.com/site/about/guidelines.xhtml

requires the child to match easily recognisable pictures (e.g. house, car, apple) and is indicated in verbal children who cannot match letter optotypes.[52] In preverbal children, we will use Keeler Acuity Cards (Keeler Ltd, Windsor, UK) for preferential looking testing. Contrast sensitivity will be measured where possible using the Thomson Test Chart contrast sensitivity module (Thomson Software Solutions, Hatfield, UK), similar to the Pelli-Robson test. Visual field testing will be performed where possible using the Octopus 900 (Haag Streit UK Ltd, Harlow, UK) and/or Humphrey Field Analyzer 3 (Carl Zeiss Ltd, Cambridge, UK). Wherever possible, VEP assessments will be performed and graded according to a modified criterion created by Thompson *et al*.[53] In children with craniosynostosis monitored at GOSH, clinical assessments and VEPs are currently performed according to an ophthalmological surveillance protocol.[54]

Sample size calculation

A cohort of at least 39 children is required to demonstrate at least 40% improvement in sensitivity compared to 22% demonstrated by fundoscopy demonstrated by Tuite *et al*[27] (McNemar's Z-test: n=39; power=80%, α=0.05). This would demonstrate sensitivity of at least 62%, a conservative estimate comparable to the least sensitive OCT measure demonstrated by Swanson *et al*[16] (maximal retinal thickness, 63% sensitivity) for detecting IH in children with craniosynostosis.

Statistical analysis

Diagnostic accuracy testing will be performed to calculate sensitivity, specificity, positive predictive value, negative predictive value and accuracy of OCT parameters to detect IH, using the OCT parameters and two definitions of IH described above. Receiver operating characteristic (ROC) curves will be plotted to evaluate diagnostic accuracy of OCT parameters for IH. Mixed model regression analysis will be performed to assess the strength of association between OCT parameters and secondary outcome measures, described above. The eye recorded (right/left) will be included in the model, to address inter-eye correlations.

Timing

Our study commenced in February 2020 but was halted in between March 2020 and September 2020 due to COVID-19 restrictions in the United Kingdom. Our study restarted in September 2020. We aim to finalise this study by February 2024.

Assessment of possible adverse effects

OCT is a safe and non-contact imaging technique that uses low-coherence light. It is extremely unlikely that any adverse events could arise during OCT imaging. Standard clinical care will continue during and beyond the completion of the RIO Study.

Patient and public involvement statement

Patients, families and the public will be actively involved in the RIO Study via two advisory groups: i) the Young Person's Advisory Group (YPAG), including participants from the GOSH YPAG and Moorfields Eye-YPAG; ii) the GOSH Parent and Carer Advisory Group (PCAG). Participants in both advisory groups will assist the RIO investigators in several ways, including but not limited to: i) sharing their lived experience of the conditions being studied; ii) keeping the study child-friendly and parent-friendly; iii) keeping recruitment rates high; iv) dissemination of study findings. Both advisory groups will meet at least annually.

For peer review only - http://bmjopen.bmj.com/site/about/guidelines.xhtml

Ethics and dissemination

Ethical approval was granted for this study by the East Midlands Nottingham 2 Research Ethics committee (UOL0348/IRAS 105137). Participants will already be under the care of GOSH, with continuity of care beyond our study. Our research clinic will involve rapid, non-invasive OCT imaging during the patients' existing appointments ± additional appointments only where required. Our study will not affect the overall management of the children. Consent will be sought from parents/guardians. Our findings will be disseminated via relevant local and national/international meetings, as well as via peer reviewed journal publication and social media.

For peer review only

For peer review only - http://bmjopen.bmj.com/site/about/guidelines.xhtml

RIO Study Protocol

Authors' contributions

SRR: Concept, protocol writing, statistical methods, final approval.

NUOJ: Concept, protocol writing, critical revision, final approval.

RB: Concept, protocol writing, critical revision, final approval.

CB: Concept, protocol writing, statistical methods, critical revision, final approval.

FAP: Concept, protocol writing, statistical methods, critical revision, final approval.

IG: Concept, protocol writing, statistical methods, critical revision, final approval.

Acknowledgements

We would like to thank the participants of GOSH Young Persons Advisory Group (YPAG), Moorfields Eye YPAG and GOSH Parent and Carer Advisory Group for supporting this study. This work is supported by the Medical Research Council, UK (Grant No. MR/N004566/1). Dr Sohaib Rufai is funded by the National Institute of Health Research Doctoral Fellowship (Award ID: NIHR300155). This work is supported by the NIHR Great Ormond Street Hospital Biomedical Research Centre. This protocol proposes independent research funded by the National Institute for Health Research (NIHR) and the Medical Research Council, UK. The views expressed are those of the author(s) and not necessarily those of the MRC, the NHS, the NIHR or the Department of Health and Social Care.

For peer review only

For peer review only - http://bmjopen.bmj.com/site/about/guidelines.xhtml

References

1. Quincke, H. Ueber Meningitis serosa und verwandte Zustände. *Deutsche Zeitschrift f. Nervenheilkunde* **9,** 149–168 (1896). https://doi.org/10.1007/BF01668270
2. Derderian C, Seaward J. Syndromic craniosynostosis. Semin Plast Surg. 2012 May;26(2):64-75. doi: 10.1055/s-0032-1320064. PMID: 23633933; PMCID: PMC3424695.
3. Jordan CO, Aylward SC. Intracranial hypertension: a current review. Curr Opin Pediatr. 2018 Dec;30(6):764-774. doi: 10.1097/MOP.0000000000000689. PMID: 30188410.
4. Aylward SC, Reem RE. Pediatric Intracranial Hypertension. Pediatr Neurol. 2017 Jan;66:32-43. doi: 10.1016/j.pediatrneurol.2016.08.010. Epub 2016 Sep 17. PMID: 27940011.
5. Matthews YY, Dean F, Lim MJ, Mclachlan K, Rigby AS, et al. Pseudotumor cerebri syndrome in childhood: incidence, clinical profile and risk factors in a national prospective population-based cohort study. Arch Dis Child. 2017 Aug;102(8):715-721. doi: 10.1136/archdischild-2016-312238. Epub 2017 Mar 29. PMID: 28356250.
6. Gillson N, Jones C, Reem RE, Rogers DL, Zumberge N, Aylward SC. Incidence and Demographics of Pediatric Intracranial Hypertension. Pediatr Neurol. 2017 Aug;73:42-47. doi: 10.1016/j.pediatrneurol.2017.04.021. Epub 2017 Apr 27. PMID: 28668233.
7. Mollan SP, Davies B, Silver NC, Shaw S, Mallucci CL, et al. Idiopathic intracranial hypertension: consensus guidelines on management. J Neurol Neurosurg Psychiatry. 2018 Oct;89(10):1088-1100. doi: 10.1136/jnnp-2017-317440. Epub 2018 Jun 14. PMID: 29903905; PMCID: PMC6166610.
8. Radhakrishnan K, Thacker AK, Bohlaga NH, Maloo JC, Gerryo SE. Epidemiology of idiopathic intracranial hypertension: a prospective and case-control study. J Neurol Sci. 1993 May;116(1):18-28. doi: 10.1016/0022-510x(93)90084-c. PMID: 8509801.
9. Raoof N, Sharrack B, Pepper IM, Hickman SJ. The incidence and prevalence of idiopathic intracranial hypertension in Sheffield, UK. Eur J Neurol. 2011 Oct;18(10):1266-8. doi: 10.1111/j.1468-1331.2011.03372.x. Epub 2011 Mar 22. PMID: 21426442.
10. Radhakrishnan K, Ahlskog JE, Cross SA, Kurland LT, O'Fallon WM. Idiopathic intracranial hypertension (pseudotumor cerebri). Descriptive epidemiology in Rochester, Minn, 1976 to 1990. Arch Neurol. 1993 Jan;50(1):78-80. doi: 10.1001/archneur.1993.00540010072020. PMID: 8418804.
11. Cinciripini GS, Donahue S, Borchert MS. Idiopathic intracranial hypertension in prepubertal pediatric patients: characteristics, treatment, and outcome. Am J Ophthalmol. 1999 Feb;127(2):178-82. doi: 10.1016/s0002-9394(98)00386-9. PMID: 10030560.
12. Kesler A, Fattal-Valevski A. Idiopathic intracranial hypertension in the pediatric population. J Child Neurol. 2002 Oct;17(10):745-8. doi: 10.1177/08830738020170101401. PMID: 12546428.
13. Babikian P, Corbett J, Bell W. Idiopathic intracranial hypertension in children: the Iowa experience. J Child Neurol. 1994 Apr;9(2):144-9. doi: 10.1177/088307389400900208. PMID: 8006364.
14. Phillips PH, Repka MX, Lambert SR. Pseudotumor cerebri in children. J AAPOS. 1998 Feb;2(1):33-8. doi: 10.1016/s1091-8531(98)90107-0. PMID: 10532364.
15. Tamburrini G, Caldarelli M, Massimi L, Santini P, Di Rocco C. Intracranial pressure monitoring in children with single suture and complex craniosynostosis: a review. Childs Nerv Syst. 2005 Oct;21(10):913-21. doi: 10.1007/s00381-004-1117-x. Epub 2005 May 3. PMID: 15871027.
16. Swanson JW, Aleman TS, Xu W, Ying GS, Pan W, et al Evaluation of Optical Coherence Tomography to Detect Elevated Intracranial Pressure in Children. JAMA Ophthalmol. 2017 Apr 1;135(4):320-328. doi: 10.1001/jamaophthalmol.2017.0025. PMID: 28241164; PMCID: PMC5470406.
17. Renier D, Sainte-Rose C, Marchac D, Hirsch JF. Intracranial pressure in craniostenosis. J Neurosurg. 1982 Sep;57(3):370-7. doi: 10.3171/jns.1982.57.3.0370. PMID: 7097333.

For peer review only - http://bmjopen.bmj.com/site/about/guidelines.xhtml

18. Wall SA, Thomas GP, Johnson D, Byren JC, Jayamohan J, et al. The preoperative incidence of raised intracranial pressure in nonsyndromic sagittal craniosynostosis is underestimated in the literature. J Neurosurg Pediatr. 2014 Dec;14(6):674-81. doi: 10.3171/2014.8.PEDS1425. Epub 2014 Sep 26. PMID: 25259602.
19. Gault DT, Renier D, Marchac D, Jones BM. Intracranial pressure and intracranial volume in children with craniosynostosis. Plast Reconstr Surg. 1992 Sep;90(3):377-81. doi: 10.1097/00006534-199209000-00003. PMID: 1513883.
20. Arnaud E, Renier D, Marchac D. Prognosis for mental function in scaphocephaly. J Neurosurg. 1995 Sep;83(3):476-9. doi: 10.3171/jns.1995.83.3.0476. PMID: 7666225.
21. Eide PK, Helseth E, Due-Tønnessen B, Lundar T. Assessment of continuous intracranial pressure recordings in childhood craniosynostosis. Pediatr Neurosurg. 2002 Dec;37(6):310-20. doi: 10.1159/000066311. PMID: 12422046.
22. Pople IK, Muhlbauer MS, Sanford RA, Kirk E. Results and complications of intracranial pressure monitoring in 303 children. Pediatr Neurosurg. 1995;23(2):64-7. doi: 10.1159/000120938. PMID: 8555097.
23. Gambardella G, Zaccone C, Cardia E, Tomasello F. Intracranial pressure monitoring in children: comparison of external ventricular device with the fiberoptic system. Childs Nerv Syst. 1993 Dec;9(8):470-3. doi: 10.1007/BF00393552. PMID: 8124675.
24. Eide PK, Helseth E, Due-Tønnessen B, Lundar T. Changes in intracranial pressure after calvarial expansion surgery in children with slit ventricle syndrome. Pediatr Neurosurg. 2001 Oct;35(4):195-204. doi: 10.1159/000050421. PMID: 11694797.
25. Hayward R, Britto J, Dunaway D, Jeelani O. Connecting raised intracranial pressure and cognitive delay in craniosynostosis: many assumptions, little evidence. J Neurosurg Pediatr. 2016 Aug;18(2):242-50. doi: 10.3171/2015.6.PEDS15144. Epub 2016 May 13. PMID: 27176895.
26. Czosnyka M, Pickard JD. Monitoring and interpretation of intracranial pressure. J Neurol Neurosurg Psychiatry. 2004 Jun;75(6):813-21. doi: 10.1136/jnnp.2003.033126. PMID: 15145991; PMCID: PMC1739058.
27. Tuite GF, Chong WK, Evanson J, Narita A, Taylor D, et al. The effectiveness of papilledema as an indicator of raised intracranial pressure in children with craniosynostosis. Neurosurgery. 1996 Feb;38(2):272-8. doi: 10.1097/00006123-199602000-00009. PMID: 8869054.
28. Tuite GF, Evanson J, Chong WK, Thompson DN, Harkness WF, Jones BM, Hayward RD. The beaten copper cranium: a correlation between intracranial pressure, cranial radiographs, and computed tomographic scans in children with craniosynostosis. Neurosurgery. 1996 Oct;39(4):691-9. doi: 10.1097/00006123-199610000-00007. PMID: 8880760.
29. Driessen C, Bannink N, Lequin M, van Veelen ML, Naus NC, et al. Are ultrasonography measurements of optic nerve sheath diameter an alternative to funduscopy in children with syndromic craniosynostosis? J Neurosurg Pediatr. 2011 Sep;8(3):329-34. doi: 10.3171/2011.6.PEDS10547. PMID: 21882928.
30. Lee H, Proudlock FA, Gottlob I. Pediatric Optical Coherence Tomography in Clinical Practice-Recent Progress. Invest Ophthalmol Vis Sci. 2016 Jul 1;57(9):OCT69-79. doi: 10.1167/iovs.15-18825. PMID: 27409508.
31. Chen CL, Bojikian KD, Xin C, Wen JC, Gupta D, et al. Repeatability and reproducibility of optic nerve head perfusion measurements using optical coherence tomography angiography. J Biomed Opt. 2016 Jun 1;21(6):65002. doi: 10.1117/1.JBO.21.6.065002. PMID: 27272095; PMCID: PMC4896902.
32. 32. Wang JK, Kardon RH, Kupersmith MJ, Garvin MK. Automated quantification of volumetric optic disc swelling in papilledema using spectral-domain optical coherence tomography. Invest Ophthalmol Vis Sci. 2012 Jun 29;53(7):4069-75. doi: 10.1167/iovs.12-9438. PMID: 22599584; PMCID: PMC3392074.

For peer review only - http://bmjopen.bmj.com/site/about/guidelines.xhtml

33. Albrecht P, Blasberg C, Ringelstein M, Müller AK, Finis D, et al. Optical coherence tomography for the diagnosis and monitoring of idiopathic intracranial hypertension. J Neurol. 2017 Jul;264(7):1370-1380. doi: 10.1007/s00415-017-8532-x. Epub 2017 Jun 5. PMID: 28584914.
34. Kaufhold F, Kadas EM, Schmidt C, Kunte H, Hoffmann J, Zimmermann H, Oberwahrenbrock T, Harms L, Polthier K, Brandt AU, Paul F. Optic nerve head quantification in idiopathic intracranial hypertension by spectral domain OCT. PLoS One. 2012;7(5):e36965. doi: 10.1371/journal.pone.0036965. Epub 2012 May 15. PMID: 22615858; PMCID: PMC3352870.
35. Rufai SR, Hisaund M, Jeelani NUO, McLean RJ. Detection of intracranial hypertension in children using optical coherence tomography: a systematic review. BMJ Open. 2021 Aug 11;11(8):e046935. doi: 10.1136/bmjopen-2020-046935. PMID: 34380720.
36. Lee H, Proudlock F, Gottlob I. Is handheld optical coherence tomography reliable in infants and young children with and without nystagmus? Invest Ophthalmol Vis Sci. 2013 Dec 17;54(13):8152-9. doi: 10.1167/iovs.13-13230. PMID: 24222299; PMCID: PMC4054919.
37. Patel A, Purohit R, Lee H, Sheth V, Maconachie G, et al. Optic Nerve Head Development in Healthy Infants and Children Using Handheld Spectral-Domain Optical Coherence Tomography. Ophthalmology. 2016 Oct;123(10):2147-57. doi: 10.1016/j.ophtha.2016.06.057. Epub 2016 Aug 9. PMID: 27521172; PMCID: PMC5036922.
38. Lee H, Purohit R, Patel A, Papageorgiou E, Sheth V, et al. In Vivo Foveal Development Using Optical Coherence Tomography. Invest Ophthalmol Vis Sci. 2015 Jul;56(8):4537-45. doi: 10.1167/iovs.15-16542. PMID: 26200492.
39. Anwar S, Nath M, Patel A, Lee H, Brown S, et al. Potential utility of fovael morphology in preterm infants measured using hand-held optical coherence tomography in retinopathy of prematurity screening. Retina. 2020 Aug;40(8):1592-1602. doi: 10.1097/IAE.0000000000002622. PMID: 31356497; PMCID: PMC7392578.
40. Rufai SR, Thomas MG, Purohit R, Bunce C, Lee H, et al. Can Structural Grading of Foveal Hypoplasia Predict Future Vision in Infantile Nystagmus?: A Longitudinal Study. Ophthalmology. 2020 Apr;127(4):492-500. doi: 10.1016/j.ophtha.2019.10.037. Epub 2019 Nov 4. PMID: 31937464; PMCID: PMC7105819.
41. Pilat A, Sibley D, McLean RJ, Proudlock FA, Gottlob I. High-Resolution Imaging of the Optic Nerve and Retina in Optic Nerve Hypoplasia. Ophthalmology. 2015 Jul;122(7):1330-9. doi: 10.1016/j.ophtha.2015.03.020. Epub 2015 May 1. PMID: 25939636; PMCID: PMC4518044.
42. Thomas MG, Kumar A, Kohl S, Proudlock FA, Gottlob I. High-resolution in vivo imaging in achromatopsia. Ophthalmology. 2011 May;118(5):882-7. doi: 10.1016/j.ophtha.2010.08.053. Epub 2011 Jan 6. PMID: 21211844.
43. Mohammad S, Gottlob I, Sheth V, Pilat A, Lee H, et al. Characterization of Abnormal Optic Nerve Head Morphology in Albinism Using Optical Coherence Tomography. Invest Ophthalmol Vis Sci. 2015 Jul;56(8):4611-8. doi: 10.1167/iovs.15-16856. PMID: 26200501.
44. Pilat AV, Proudlock FA, Shah S, Sheth V, Purohit R, et al. Assessment of the anterior segment of patients with primary congenital glaucoma using handheld optical coherence tomography. Eye (Lond). 2019 Aug;33(8):1232-1239. doi: 10.1038/s41433-019-0369-3. Epub 2019 Mar 18. PMID: 30886322; PMCID: PMC7005739.
45. Papageorgiou E, Pilat A, Proudlock F, Lee H, Purohit R, et al. Retinal and optic nerve changes in microcephaly: An optical coherence tomography study. Neurology. 2018 Aug 7;91(6):e571-e585. doi: 10.1212/WNL.0000000000005950. Epub 2018 Jul 11. PMID: 29997194; PMCID: PMC6105049.
46. Rufai SR, Gore S, Handley SE, Marmoy OR, Ong J, et al. Enhanced neuro-ophthalmologic evaluation to support separation of craniopagus twins. J Surg Case Rep. 2021 Feb 4;2021(2):rjaa606. doi: 10.1093/jscr/rjaa606. PMID: 33569170; PMCID: PMC7861574.
47. Rufai SR, Bowman R, Bunce C, Panteli V, McLean RJ, et al. Feasibility and Repeatability of Handheld Optical Coherence Tomography in Children With Craniosynostosis. Transl Vis Sci

For peer review only - http://bmjopen.bmj.com/site/about/guidelines.xhtml

Technol. 2021 Jul 1;10(8):24. doi: 10.1167/tvst.10.8.24. PMID: 34313724; PMCID: PMC8322722.

48. Schneider C, Rasband W, Eliceiri K. NIH Image to ImageJ: 25 years of image analysis. Nat Methods. 2012; 9: 671–675.

49. ImageJ. ImageJ Documentation Wiki: active contour (snake). Available at: https://imagejdocu.tudor.lu/plugin/segmentation/active_contour/start. Accessed 20 August, 2021.

50. Maldonado RS, Izatt JA, Sarin N, et al. Optimizing hand-held spectral domain optical coherence tomography imaging for neonates, infants, and children. Invest Ophthalmol Vis Sci. 2010; 51(5): 2678–2685.

51. Shah SD, Haq A, Toufeeq S, Tu Z, Edawaji B, et al. Reliability and Recommended Settings for Pediatric Circumpapillary Retinal Nerve Fiber Layer Imaging Using Hand-Held Optical Coherence Tomography. Transl Vis Sci Technol. 2020 Jun 30;9(7):43. doi: 10.1167/tvst.9.7.43. PMID: 32832248; PMCID: PMC7414610.

52. O'Boyle C, Chen SI, Little JA. Crowded letter and crowded picture logMAR acuity in children with amblyopia: a quantitative comparison. Br J Ophthalmol. 2017 Apr;101(4):457-461. doi: 10.1136/bjophthalmol-2015-307677. Epub 2016 Jul 7. PMID: 27388249; PMCID: PMC5583677.

53. Thompson DA, Liasis A, Hardy S, Hagan R, Hayward RD, et al. Prevalence of abnormal pattern reversal visual evoked potentials in craniosynostosis. Plast Reconstr Surg. 2006 Jul;118(1):184-92. doi: 10.1097/01.prs.0000220873.72953.3e. PMID: 16816694.

54. Hinds AM, Thompson DA, Rufai SR, Weston K, Schwiebert K, et al. Visual outcomes in children with syndromic craniosynostosis: a review of 165 cases. Eye (Lond). 2021 May 10. doi: 10.1038/s41433-021-01458-5. Epub ahead of print. PMID: 33972704.

For peer review only

For peer review only - http://bmjopen.bmj.com/site/about/guidelines.xhtml
